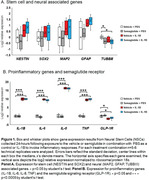# Oral Concurrent Session 4 – Basic and Translational Science: Friday, January 31, 2025 1:30 PM – 4:00 PM

**DOI:** 10.1002/pmf2.12012

**Published:** 2025-01-05

## 47 | Novel Neural Extracellular Vesicle miRNA Biomarkers Identify Suboptimal Response to Cooling in Neonatal Encephalopathy

Sarah T. Mehl^1^; Baharan Fekry^1^; Lierni Ugartemendia^1^; Dhanashree Rajderkar^2^; Nikolay Bliznyuk^2^; Shaveka Gaskins^2^; Michael D. Weiss^3^; Laura Goetzl^1^

*^1^McGovern Medical School at UTHealth Houston, Houston, TX; ^2^University of Florida, Gainesville, FL; ^3^University of Florida College of Medicine, Gainesville, FL*

1:30 PM ‐ 1:45 PM

**Objective**: Recent research has identified multiple circulating miRNA biomarkers that may predict neonatal brain injury following hypoxia‐ischemia. We hypothesized that quantification of these miRNAs in CNS derived extracellular vesicles (EV) would result in more accurate prediction of subsequent brain injury given that total circulating markers represent miRNAs from multiple organ sources.

**Study Design**: CNS EVs were purified from serum samples from 35 neonates who underwent therapeutic hypothermia for NE using previously published methods. Neonates were subsequently categorized into 2 groups based on post warming MRI using the Barkovich scoring system: no/mild injury (n = 21) or moderate/severe injury (n = 14). The four most predictive circulating miRNAs were selected and quantified in CNS EVs using standard qPCR techniques at two time points after birth: 0‐6 hours and 48 hours. Biomarker levels were compared between no/mild and moderate/severe injury using the Kruskal‐Wallis test and ROC curves were constructed for individual and combined miRNAs.

**Results**: All miRNA biomarkers were significantly different between groups at 48 hours, but miRNAs 328‐3p and 342‐3p were the most effective at discriminating between the two clinical outcomes (Figure 1a, miRNAs 197‐3p and 150‐5p not shown). The combination of all 4 miRNAs resulted in an area under the curve of 0.96 (Figure 1b). However, miRNA 328‐3p as a single marker had a similar performance (AUC 0.93) and may be a more cost‐effective approach.

**Conclusion**: Predictive biomarkers are critically important in identifying neonates who will have residual neurologic injury following neonatal encephalopathy despite therapeutic hypothermia. Early identification would allow for early implementation of additional neuroprotective interventions in this subpopulation. We present data identifying 1 to 4 miRNAs that appear to be effective biomarkers at 48 hours. Further investigation is required to determine if CNS EV miRNAs are superior to circulating miRNAs in this clinical scenario.

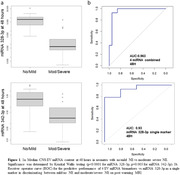

## 48 | 3D‐Printed Humanized Feto‐Maternal Interface Tests Exosomal Delivery of Anti‐Inflammatory Interleukin‐10 (IL‐10) to Reduce Infection‐Associated Inflammation

Leah Saylor^1^; Rahul Cherukuri^2^; Ananth Kammala^1^; Marc Ferrer^3^; Cristina Antich Acedo^3^; Arum Han^2^; Lauren Richardson^1^; Ramkumar Menon^1^

*^1^University of Texas Medical Branch, Galveston, TX; ^2^Texas A&M University, College Station, TX; ^3^National Center for Advancing Translational Sciences, National Institute of Sciences, Bethesda, MD*

1:45 PM ‐ 2:00 PM

**Objective**: Spontaneous preterm birth (PTB) is linked to fetal inflammatory responses (FIR), typically caused by infections. Current treatments are limited because drugs often cannot cross the feto‐maternal interface (FMi). This study uses New Approach Methods (NAMs), like extracellular vesicles (EVs) for drug delivery and a high‐throughput 3D‐printed organ‐on‐chip (OOC) model to test efficacy.

**Study Design**: Recombinant IL‐10 was delivered in extracellular vesicles (eIL‐10) via electroporation and through THP1 cells transfected with an IL‐10 plasmid (tIL‐10). A two‐chambered 3D scaffold mimicking the feto‐maternal interface was used for high‐throughput screening. The scaffold, made from biocompatible polymer resin via 3D printing, features microchannels for fluidic communication and a mix of cells and gelatin methacrylate hydrogel in the lower chamber, with cell‐specific medium in the upper chamber. We assessed cell viability (LDH), metabolic activity (AlamarBlue), and cytoskeletal markers (cytokeratin‐18 and vimentin). Maternal infection was simulated using Lipopolysaccharide (LPS), and the efficacy of eIL‐10 and tIL‐10 (500ng/mL) in reducing inflammation was evaluated by measuring IL‐6 and IL‐8 concentrations at 6 and 24 hours.

**Results**: Our 3D‐printed OOC FMi model supported cell growth, viability, and metabolic activity. LPS, eIL‐10, and tIL‐10 successfully moved between the interconnected chambers. LPS increased IL‐6 (p< 0.001) and IL‐8 (p< 0.001) in both decidua and amnion compared to controls. Co‐treatment with IL‐10, in any form, significantly reduced cytokines on both maternal (p< 0.05) and fetal sides (p< 0.05).

**Conclusion**: We developed a 3D‐printed feto‐maternal interface that effectively tested and showed that exosomal encapsulated IL‐10 reduces FMi infectious inflammation. We propose two NAMs: (1) exosomal drug delivery and (2) a 3D‐printed device for high‐throughput drug screening. These approaches could significantly improve perinatal medicine by reducing inflammation that leads to preterm birth.

## 49 | Decoy Peptide to (pro)renin Receptor as a Potential Therapeutic Target for Preeclampsia: a Translational Study

Michelle Harris^1^; Ahmed F. Pantho^2^; Kelsey R. Kelso^1^; Jessica C. Ehrig^3^; Ram R. Kalagiri^1^; Niraj Vora^1^; Thomas J. Kuehl^2^; Steven R. Lindheim^1^; Mohammad N. Uddin^4^

*^1^Baylor Scott & White Health Temple Medical Center, Temple, TX; ^2^Artemis Biotechnologies LLC, Temple, TX; ^3^Baylor Scott and White Health, Temple, TX; ^4^Baylor Scott & White Medical Center and Texas A&M School of Medicine, Temple, TX*

2:00 PM ‐ 2:15 PM

**Objective**: Preeclampsia (preE), a syndrome of hypertension, proteinuria, and end organ damage, is a leading cause of maternal and fetal morbidity and mortality. Circulating levels of soluble (pro)renin receptor (PRR) and placental (pro)renin (PR) have been shown to be elevated in a preE rat model and preE human subjects with a decoy peptide based on this handle‐region peptide (HRP) that can block binding of PR to PRR. The goal of this study was to develop and characterize HRP as an innovative treatment for preE and PRR as a novel biomarker for preE.

**Study Design**: The human extravillous cytotrophoblasts (CTBs) incubated with PRR in medium with the receptor‐expressing CTBs followed by renin activity assay to determine the percent binding and activation of PRR. Renin activity assay was performed to determine the percent binding and activation of PRR. Pregnant female rats were randomly assigned to three pregnant groups: 1) NP: normal rats; 2) PDS: rats injected desoxycorticosterone acetate (DOCA) whose drinking water was replaced with saline; and 3) PDS‐HRP: rats administered DOCA and saline and given HRP during pregnancy. Additionally, placental and blood samples were collected from pregnant women (40 NP and 30 preE) and analyzed by WB and IHC and s(P)RR ELISA in an IRB approved study.

**Results**: PR was activated by binding to PRR on the cell membrane of CTB cells, while HRP inhibited binding (100% vs 20%, p< 0.05) and activation of PRR (100% vs 30%, p< 0.05). HRP administration normalized blood pressure, proteinuria, and birth numbers in the DOCA preE rat model. The placental expression of PRR and soluble PRR were higher (p< 0.05) in human preE compared to NP women, respectively.

**Conclusion**: Our data suggests that increased expression of PRR in the placenta is associated with elevated levels of soluble PRR and is related to preE in the rat model and human subjects. Given HRP appears to inhibit binding of PR to PRR, its use may serve as a potential therapeutic intervention in preE.

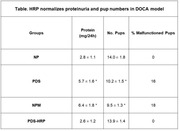

## 50 | Longitudinal Changes in Epigenetic Aging in Pregnant Versus Non‐Pregnant People: a Prospective Cohort Study

Danielle M. Panelli; Nicole Gladish; Nicola C. Perlman; Andres Cardenas; Katherine Bianco

*Stanford University, Palo Alto, CA*

2:15 PM ‐ 2:30 PM

**Objective**: Higher parity has been linked with accelerated biologic aging, yet the direct effects of pregnancy itself on epigenetic aging biomarkers are unknown. We compared changes in epigenetic aging across gestation between pregnant and non‐pregnant people.

**Study Design**: This was a prospective cohort of nulliparous people aged 18‐50 years seeking obstetric or gynecologic care in 2020. Pregnant people were enrolled between 10 and 14 weeks gestation and age‐matched to the next eligible non‐pregnant person. Blood samples were collected at enrollment (Time 1) and again either on postpartum day 1 (pregnant) or approximately 7 months later (non‐pregnant, Time 2). The primary outcome was biological age, determined from epigenetic clocks using Illumina EPIC Human Methylation BeadChips. Biologic ages at specific time points were compared using t‐tests. Changes in biologic age over time were analyzed using mixed effects linear regression models, adjusting for confounders and interaction between time and pregnancy status. Results focus on the two strongest clocks: GrimAge (biologic age in years) and DunedinPACE [aging acceleration (> 1) or deceleration (< 1)].

**Results**: 75 people enrolled, of whom 61 (81.3%) completed both timepoints. While the sample interval differed by cohort, their chronologic ages were similar (Table 1). By Time 2, the pregnant cohort had an older GrimAge than the non‐pregnant cohort [mean (SD) 56.7 (3.5) versus 52.9 (6.0) years, p = < 0.01). Additionally, DunedinPACE indicated faster aging in the pregnant cohort at Time 2 [1.2 (0.1) pregnant versus 1.0 (0.1) non‐pregnant, p = < 0.001]. Significant interaction between time and pregnancy status was detected for both GrimAge (p< 0.01) and DunedinPACE (p< 0.01, Figure 1).

**Conclusion**: We found significant epigenetic aging acceleration in nulliparous pregnant people compared to their non‐pregnant counterparts, highlighting gestation as a key period for epigenetic changes. Further characterizing these epigenetic aging trajectories may inform future interventions to improve maternal and child health.

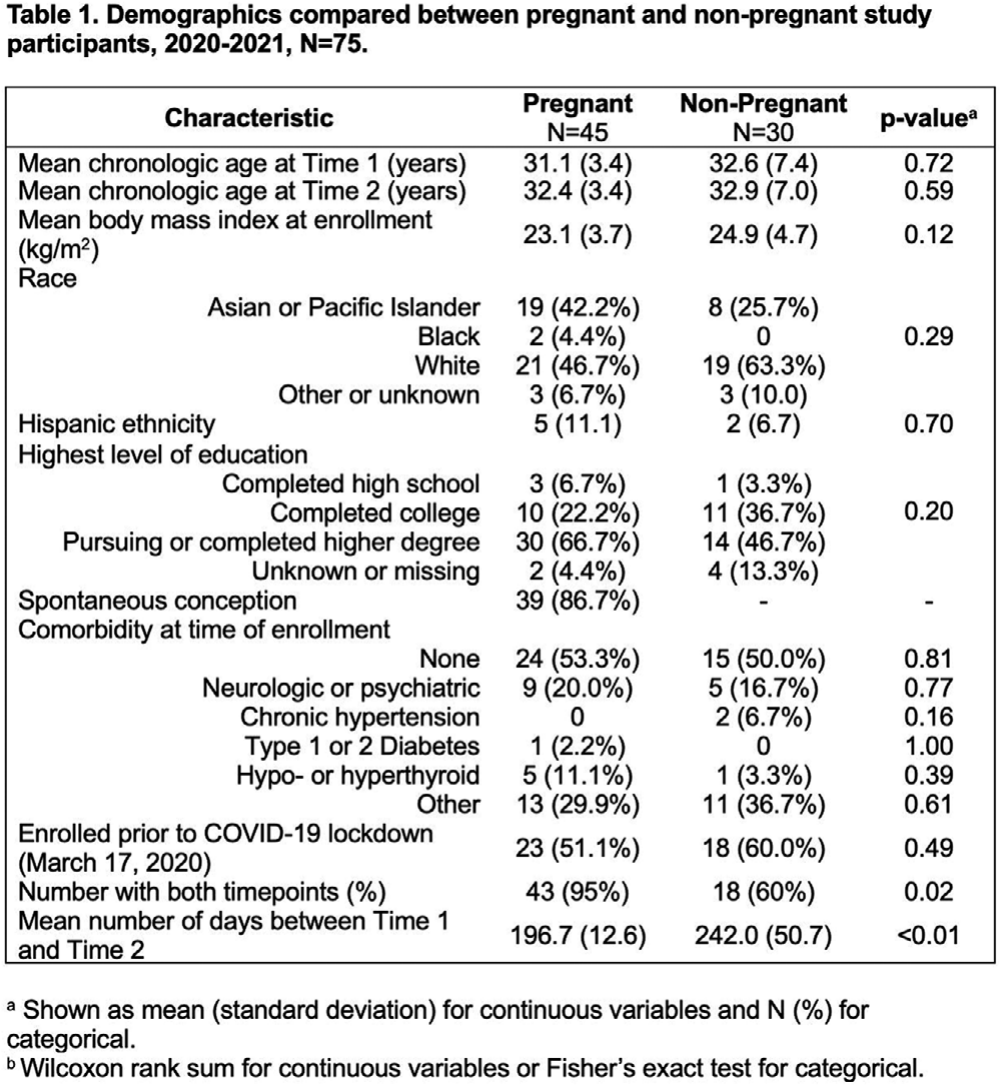

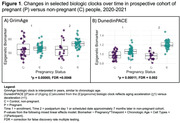

## 51 | Lactobacillus Crispatus Mitigates Group B Streptococcus‐Induced Inflammation in the Cervicovaginal Epithelium

Briana Ferguson; Aaron Loder; Lauren Anton; Kristin D. Gerson

*University of Pennsylvania Perelman School of Medicine, Philadelphia, PA*

2:30 PM ‐ 2:45 PM

**Objective**: Group B *Streptococcus* (GBS) is a common vaginal microbe involved in adverse perinatal outcomes, including preterm birth, chorioamnionitis, and neonatal sepsis. Features of vaginal ecosystems may modify GBS pathogenicity. Previous work has shown that *Lactobacillus crispatus* (LC), a marker of reproductive health, mitigates proinflammatory cytokines in response to select vaginal pathogens. We sought to investigate the effect of LC on GBS‐mediated inflammation in the cervicovaginal epithelium.

**Study Design**: We leveraged an *in vitro* epithelial‐microbial co‐culture model. Ectocervical, endocervical, and vaginal epithelial cells were treated with 10^5^ CFUs of LC for 24h. Cells were then treated with 10^4^ CFUs of GBS clinical isolates (serotypes IA, III, and IV) for an additional 24h. Concentration of proinflammatory cytokine IL‐8 was measured in cell culture media by ELISA. Cytotoxicity was assessed using LDH assays. Outcomes were compared by one‐way ANOVA with correction for multiple comparisons. Microbial count (CFU/well) was measured on De Man‐Rogosa‐Sharpe and Granada agar plates to select for GBS growth.

**Results**: LC had no effect on IL‐8 expression, while all GBS serotypes induced IL‐8 across cells (p < 0.05 for all, Fig. 1). LC rescued GBS induction of IL‐8 to near basal levels (p < 0.05 for all, Fig. 1). LC did not alter LDH in GBS‐treated cells (data not shown), indicating that IL‐8 reduction was not attributable to cell death. To test if immune mitigation occurred due to LC outcompeting GBS growth, we assessed microbial counts at the time of IL‐8 measurement. LC did not alter the number of viable GBS colonies, and unexpectedly no live LC was detected (Table 1).

**Conclusion**: LC mitigates host inflammatory response to a vaginal microbe implicated in adverse pregnancy outcomes. Microbial competition does not appear to underlie this protective effect, suggesting that immune‐mediated mechanisms are at play. Whether LC microbial components or secreted factors, such as metabolites, modify GBS‐epithelial interactions warrants future investigation. University of Pennsylvania Research Foundation Award (KDG)

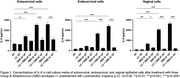

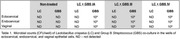

## 52 | Expression of CD300E in Human Myometrium and its Impact on Parturition

Meera M. Thakkar^1^; William E. Ackerman, IV^1^; Guomao Zhao^1^; Zoe B. Strong^1^; Elizabeth Feoktistov^2^; Ekaterina Snegovskikh^2^; Catalin S. Buhimschi^1^; Irina A. Buhimschi^1^

*^1^University of Illinois at Chicago, College of Medicine, Chicago, IL; ^2^University of Illinois at Chicago, Chicago, IL*

2:45 PM ‐ 3:00 PM

**Objective**: CD300 glycoproteins are a newly identified family of 7 receptors with paired inhibitory and activating cellular functions. CD300 receptors have recently emerged as therapeutic targets. CD300E is expressed in myocardial smooth muscle and has been linked to cardiac arrythmia. Our novel study sought to examine if CD300E is expressed in the human myometrium and if its levels of expression vary in relationship to the uterine contractile status.

**Study Design**: RNA sequencing was performed on myometrial biopsies of 22 patients in the following clinical groups (dataset GSE163773): 1) spontaneous preterm birth (PTB) with clinical and/or histologic chorioamnionitis (HCA, n = 6, GA 29±3w); 2) PTB without labor (PTNL, n = 6, GA 29±3w); 3) term birth in absence of labor (TNL, n = 5, GA: 39±1w) and 4) spontaneous term labor (TL, n = 5, GA 40±1w). An external RNAseq dataset (GSE114691) was used for validation. The combined dataset comprised 20 term and 12 preterm samples further stratified by uterine contractile status as quiescent (Q, n = 19) and non‐quiescent (NQ, n = 13). qPCR and immunohistochemistry (IHC) for CD300E were performed for confirmation.

**Results**: *CD300E* mRNA was significantly upregulated in NQ vs. Q myometrium in both datasets (Fig. **A**). This was confirmed by qPCR (Fig. **B**), which was strongly correlated with the RNAseq expression data in matched samples (r = 0.8, p< 0.001). Moreover, *CD300E* expression was significantly correlated with *ATP2B4*/*ATP2A2*, a previously characterized ratio of Ca^2+^ transporters genes used for molecular classification of myometrial contractile status (Fig. **C**), independent of GA, amniotic fluid culture results or membrane status. By IHC we validated CD300E was present in myometrial cells with a stronger labeling in labor (Fig. **D**).

**Conclusion**: CD300E is expressed in human myometrium and significantly upregulated in laboring myometrium. CD300E may prove to be future therapeutic targets for prevention of preterm birth.

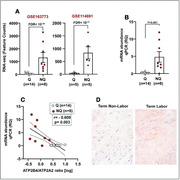

## 53 | Personalized Models of fetal Brain Development as Biomarkers of Offspring Neurodevelopmental Outcomes After Maternal SARS‐CoV‐2

Lydia L. Shook; Liam T. McCrea; Olyvia Jasset; Steven D. Sheridan; Roy H. Perlis; Andrea G. Edlow

*Massachusetts General Hospital, Boston, MA*

3:00 PM ‐ 3:15 PM

**Objective**: Maternal immune activation from viral infection in pregnancy is associated with adverse neurodevelopmental (ND) outcomes in offspring, mediated at least in part by *in utero* programming of fetal brain microglia resulting in aberrant synaptic pruning. We have shown the feasibility of using human cord blood mononuclear cells (CB‐MNCs) to model the impact of maternal viral infection on fetal brain development. In a large cohort, we examined associations between CB‐MNC models and offspring adverse ND outcomes at 3 years of age using linked electronic health records (EHR).

**Study Design**: CB‐MNCs were selected from 119 unvaccinated individuals enrolled in a COVID‐19 biorepository (April 2020 ‐ August 2022): 74 with maternal SARS‐CoV‐2 infection in pregnancy (N = 38 1^st^ or 2^nd^ trimester infection, N = 36 3^rd^ trimester infection) and 45 uninfected controls. We induced CB‐MNCs to acquire microglia‐like morphology and function (cord blood induced microglia or CB‐iMG) using IL‐34, GM‐CSF, and serum withdrawal. CB‐iMG phagocytosis of iPSc‐derived neural synaptosomes‐a proxy for microglial synaptic pruning behavior‐was quantified using real‐time pHrodo fluorescence‐based imaging. ND outcomes of offspring at 3 years were determined using an EHR‐based algorithm to identify ICD‐10 codes associated with ND in an integrated statewide health system.

**Results**: CB‐iMG exhibited typical microglial morphology and marker expression (Fig 1A). Synaptosome phagocytosis was greater (p< 0.001) in the CB‐iMG of offspring exposed to 3^rd^ trimester maternal SARS‐CoV‐2 with an adverse ND outcome (N = 6) compared to exposed offspring with no ND outcome (N = 30) (Fig 1B). Among unexposed offspring (N = 45), phagocytosis scores between groups were not significantly different. Phagocytosis in models derived from 1^st^ and 2^nd^ trimester infection did not differ significantly from controls.

**Conclusion**: Although additional validation is needed, personalized models using CB‐MNCs available at birth show promise for identifying offspring at greatest ND risk after exposure to maternal viral infection *in utero*.

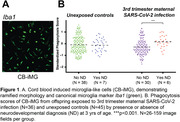

## 54 | The Placental Vascular Transcriptome in Pregnancies Resulting in Low vs. Normal Birth Weight Neonates

Samantha Carson^1^; Morgan E. Wasickanin^1^; Emily Sheikh^1^; Hillary Kinsman^1^; Lydia Bettridge^1^; Katherine E. Free^1^; Jennifer Damicis^1^; Robert Walton^1^; Peter Napolitano^2^; Nicholas Ieronimakis^1^

*^1^Madigan Army Medical Center, Tacoma, WA; ^2^University of Washington, Seattle, WA*

3:15 PM ‐ 3:30 PM

**Objective**: Placental vascular dysfunction may be associated with fetal growth restriction (FGR). Although not all Low Birth Weight (LBW) neonates are identified as FGR in the prenatal period, it is believed that abnormal placental blood flow is a contributing factor. We hypothesize that molecular differences within placental vessels underly vascular impairment in LBW neonates without known FGR etiologies (e.g. genetic or other congenital anomalies). The objective of this pilot study was to compare the vascular transcriptome within placentas delivered from normal birth weight neonates to those born with LBW.

**Study Design**: Chorionic arterial and venous vessels were dissected from unlabored placentas delivered by cesarean (Figure 1). Vessels were stripped to remove the membrane and plate along with any blood. Gene expression analysis from normal (n = 8) and LBW neonates (n = 10) was performed using mRNA‐seq. Alignment and differential gene expression (DEGs) analysis was conducted using Illumina's Dragen software. We considered DEGs significant based on a Benjamini‐Hochberg FDR adjusted p‐value of < 0.05.

**Results**: Between LBW vs. normal, 15,204 DEGs were identified in arteries and 14,667 in veins. Among these, 5 were significantly upregulated and 1 downregulated in LBW arteries (Figure 2). In LBW veins, 1 DEG was significantly upregulated (not shown). Notably, zinc finger protein (ZNF)365 was downregulated in LBW arteries while ZNF358 was upregulated in veins.

**Conclusion**: The vascular transcriptome in placental vessels from LBW neonates appears different from those with normal birth weights. Expression of ZNF365 is lower in the placental arteries of LBW baby's and ZNF358 is greater in placental veins of LBW babies. ZNF365 is involved in DNA repair and in colon cancer its correlated with poor prognosis. Little is known about ZNF358 but it may be related to vascular dysfunction. Further investigation is warranted to understand the role of these factors in regulating vascular function in relation to fetal growth.

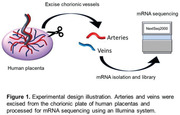

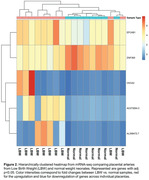

## 55 | Commonly Used Non‐Steroidal Anti‐Inflammatory Drug (NSAIDs) in Obstetrics & Associated Placental Cytotoxicity

Ana Collins‐Smith

*University of Texas Medical Branch, Galveston, TX*

3:30 PM ‐ 3:45 PM

**Objective**: To determine if commonly used non‐steroidal anti‐inflammatory drugs have a cytotoxic effect and their ability to reduce inflammation at the cellular level of the feto‐maternal interface.

**Study Design**: A microfluidic fetal membrane‐placental feto‐maternal interface on‐chip (FMi‐PLA‐OOC) composed of seven different cell types was used to analyze the effects of cell cytotoxicity at the placental‐maternal interface with therapeutic concentrations of commonly used NSAIDs in obstetrics. Therapeutic concentrations of aspirin (2µg/mL corresponding to 81mg) and indomethacin (15µg/mL) were applied to the FMi‐PLA‐OOC with the supernatants analyzed for LDH levels and cytokine levels in both a diseased state, non‐diseased state (simulated with lipopolysaccharide (LPS)), and a diseased state with treatment of the NSAIDs.

**Results**: Cell cytotoxicity in the control group was approximately 20% in AEC, AMC, CTC, and STB cell lines. After application of LPS, the cell cytotoxicity increased in five cell types. Aspirin decreased cell death by approximately 84% in AEC & 48.7% in STB. While indomethacin increased cell death across all cell lines. A three‐way ANOVA was conducted with p‐value < 0.0001. Pro‐inflammatory cytokine IL‐8 was decreased with aspirin use.

**Conclusion**: Indomethacin demonstrates cell cytotoxicity at all layers of the feto‐maternal interface. On the other hand, aspirin demonstrated an improvement in cell death in a diseased state which can support the continued use of aspirin for the promotion of placental angiogenesis. Aspirin also demonstrated a decrease in pro‐inflammatory cytokines at the placental cellular level.

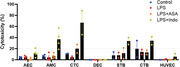

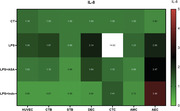

## 56 | Evaluating Semaglutide in Human Fetal Neural Stem Cells: Could Early Exposure Impact Neurodevelopment?

Morgan E. Wasickanin^1^; Samantha Carson^1^; Hillary Kinsman^1^; Katherine E. Free^1^; Jennifer Damicis^1^; Emily Sheikh^1^; Robert Walton^1^; Irina Burd^2^; Peter Napolitano^3^; Nicholas Ieronimakis^1^

*^1^Madigan Army Medical Center, Tacoma, WA; ^2^University of Maryland, Baltimore, MD; ^3^University of Washington, Seattle, WA*

3:45 PM ‐ 4:00 PM

**Objective**: Semaglutide (Ozempic/Wegovy) use for diabetes and weight loss management has exploded, especially in reproductive age females. Due to its long half‐life and possibility of crossing the placenta, discontinuation of use is recommended prior to conception. Anecdotally, unintentional pregnancies with semaglutide appear to be on the rise. Semaglutide may signal within the adult brain and reduce neuroinflammation, yet it remains unknown if this occurs in the fetal brain. We hypothesize semaglutide exposure early in pregnancy may impact neurodevelopment and/or infer anti‐inflammatory properties. We examined if semaglutide alters the phenotype and proinflammatory response of human fetal derived neural stem cells (NSCs) *in‐vitro*.

**Study Design**: Commercially available fetal NSCs (ReNcells) were exposed to either semaglutide or the vehicle. In parallel they were given PBS as a control or Interleukin‐1 beta (IL‐1B) to stimulate inflammatory responses. Cells were harvested 24‐hours post exposure (n = 5‐6 per condition) and examined for gene expression.

**Results**: The expression of genes associated with stem cell maintenance (NESTIN, SOX2) and neural differentiation (MAP2, GFAP) was not different with semaglutide exposure alone. The neuronal gene Beta‐III‐tubulin (TUBBIII) was significantly downregulated with semaglutide alone and in combination with IL‐1B (Fig. 1A). Semaglutide did not impact the upregulation of proinflammatory genes by IL‐1B. Though IL‐1B exposure did downregulate the expression of the signaling receptor (GLP‐1R), this was not related to semaglutide exposure (Fig. 1B).

**Conclusion**: Semaglutide impacts the NSCs phenotype by downregulating TUBBIII. Beta‐III‐tubulin plays an important role in neurodevelopment and has been implicated across a spectrum of long‐term brain disorders. The clinical significance of these findings are unclear but support the need for further research and careful monitoring in exposed offspring.